# Supplementary material for: Uncertainty in El Niño-like warming and California precipitation changes linked by the Interdecadal Pacific Oscillation
Source: Nat Commun. 2021 Nov 10;12:6484. doi: 10.1038/s41467-021-26797-5 (PMC8581011; doi:10.1038/s41467-021-26797-5)
Supplement: Supplementary file 1 — Supplementary Information [file 41467_2021_26797_MOESM1_ESM.pdf]

Supplementary Material

Uncertainty in El Niño-like warming and California precipitation changes linked by the Interdecadal Pacific Oscillation

Lu Dong[1], L. Ruby Leung[1], Fengfei Song[1], and Jian Lu[1]

1 Atmospheric Sciences and Global Change Division, Pacific Northwest National Laboratory, Richland, Washington, USA

Submitted to ***Nature Communications***

Contents of this file

Supplementary Table 1 to 2

Supplementary Figures 1 to 13

Corresponding authors:

Lu Dong (lu.dong@pnnl.gov) and L. Ruby Leung (ruby.leung@pnnl.gov)

Supplementary Table 1 The CMIP5 and CMIP6 models and the numbers of each simulation used in this study.

No.	CMIP5 Model	Historical	RCP8.5	CMIP6 Models	Historical	SSP585
1	ACCESS1-0	1	1	ACCESS-CM2	3	3
2	ACCESS1-3	1	1	ACCESS-ESM1-5	3	3
3	bcc-csm1-1	1	1	AWI-CM-1-1-MR	1	1
4	bcc-csm1-1-m	1	1	BCC-CSM2-MR	1	1
5	BNU-ESM	1	1	CAMS-CSM1-0	1	1
6	CanESM2	3	3	CanESM5	3	3
7	CCSM4	3	3	CESM2	1	1
8	CESM1-BGC	1	1	CESM2-WACCM	3	3
9	CESM1-CAM5	3	3	CMCC-CM2-SR5	1	1
10	CESM1-WACCM	1	1	CNRM-CM6-1	3	3
11	CMCC-CESM	1	1	CNRM-CM6-1-HR	1	1
12	CMCC-CM	1	1	CNRM-ESM2-1	3	3
13	CMCC-CMS	1	1	E3SM-1-1	1	1
14	CNRM-CM5	3	3	EC-Earth3	1	1
15	CSIRO-Mk3-6-0	3	3	EC-Earth3-Veg	3	3
16	FGOALS-g2	1	1	EC-Earth3-Veg-LR	3	3
17	FIO-ESM	1	1	FGOALS-f3-L	1	1
18	GFDL-CM3	1	1	FGOALS-g3	3	3
19	GFDL-ESM2G	1	1	FIO-ESM-2-0	3	3
20	GFDL-ESM2M	1	1	GFDL-ESM4	1	1
21	GISS-E2-H	1	1	GISS-E2-1-G	1	1
22	GISS-E2-R	1	1	HadGEM3-GC31-LL	3	3
23	HadGEM2-AO	1	1	HadGEM3-GC31-MM	3	3
24	HadGEM2-CC	3	3	IITM-ESM	1	1
25	HadGEM2-ES	3	3	INM-CM4-8	1	1
26	inmcm4	1	1	INM-CM5-0	1	1
27	IPSL-CM5A-LR	3	3	IPSL-CM6A-LR	3	3

28	IPSL-CM5A-MR	1	1	KACE-1-0-G	1	1
29	IPSL-CM5B-LR	1	1	MCM-UA-1-0	1	1
30	MIROC5	3	3	MIROC6	3	3
31	MIROC-ESM-CHEM	1	1	MIROC-ES2L	3	3
32	MIROC-ESM	1	1	MPI-ESM1-2-HR	1	1
33	MPI-ESM-LR	3	3	MPI-ESM1-2-LR	3	3
34	MPI-ESM-MR	1	1	MRI-ESM2-0	1	1
35	MRI-CGCM3	1	1	NESM3	1	1
36	NorESM1-M	1	1	TaiESM1	1	1
37	NorESM1-ME	1	1	UKESM1-0-LL	3	3

Supplementary Table 2 Percentage of the standard deviation of CA precipitation trends reduced by removing the IPO's influence.

	CESM1	CanESM2	MPI-ESM
1979-2019	0.3%	16%	12%
2020-2060	26%	11%	16%
2061-2099	12%	25%	10%

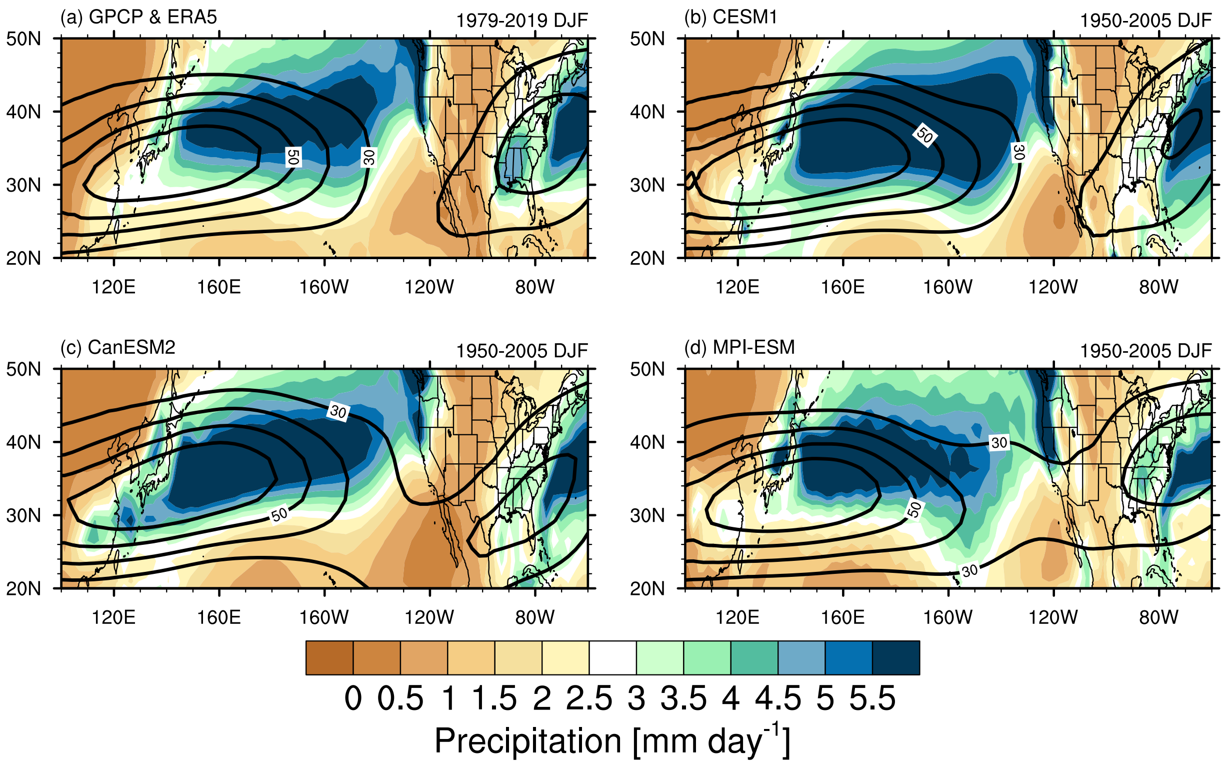

Supplementary Fig. 1 Spatial patterns of the climatological precipitation (mm day^{-1}, color) and 200hPa zonal wind (m s^{-1}, contours) in winter based on (a) observation from GPCP and ERA5, (b) 40-member mean of CESM1, (c) 50-member mean of CanESM2, (d) 100-member mean of MPI-ESM.

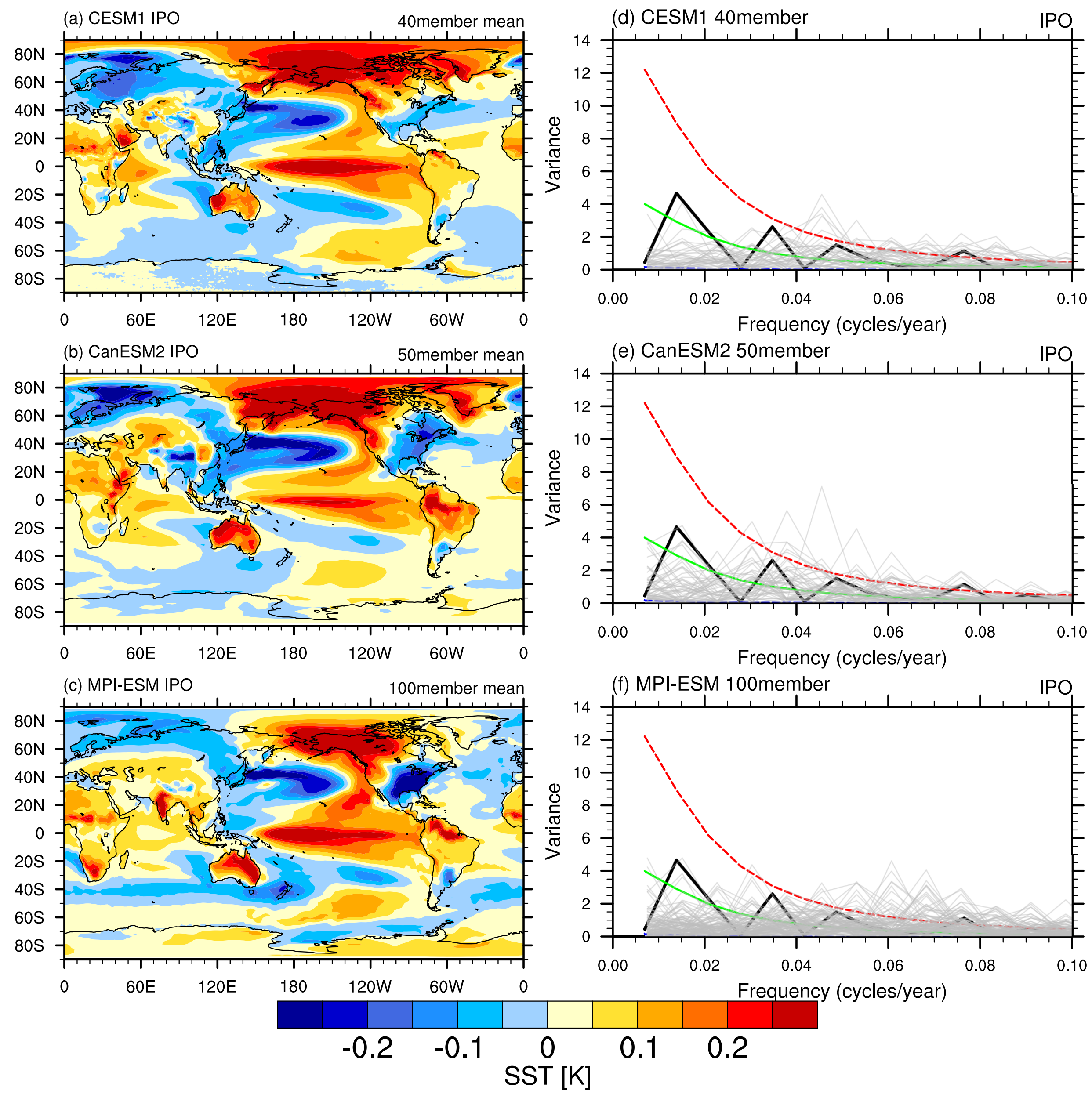

Supplementary Fig. 2 Spatial patterns and spectra analysis of the IPO index in observation from HadISST (black lines) and simulated by the three large ensembles (grey lines). The spatial patterns in (a-c) are based on the mean of all the members of a given large ensemble, and each grey line in (d-f) represents one member.

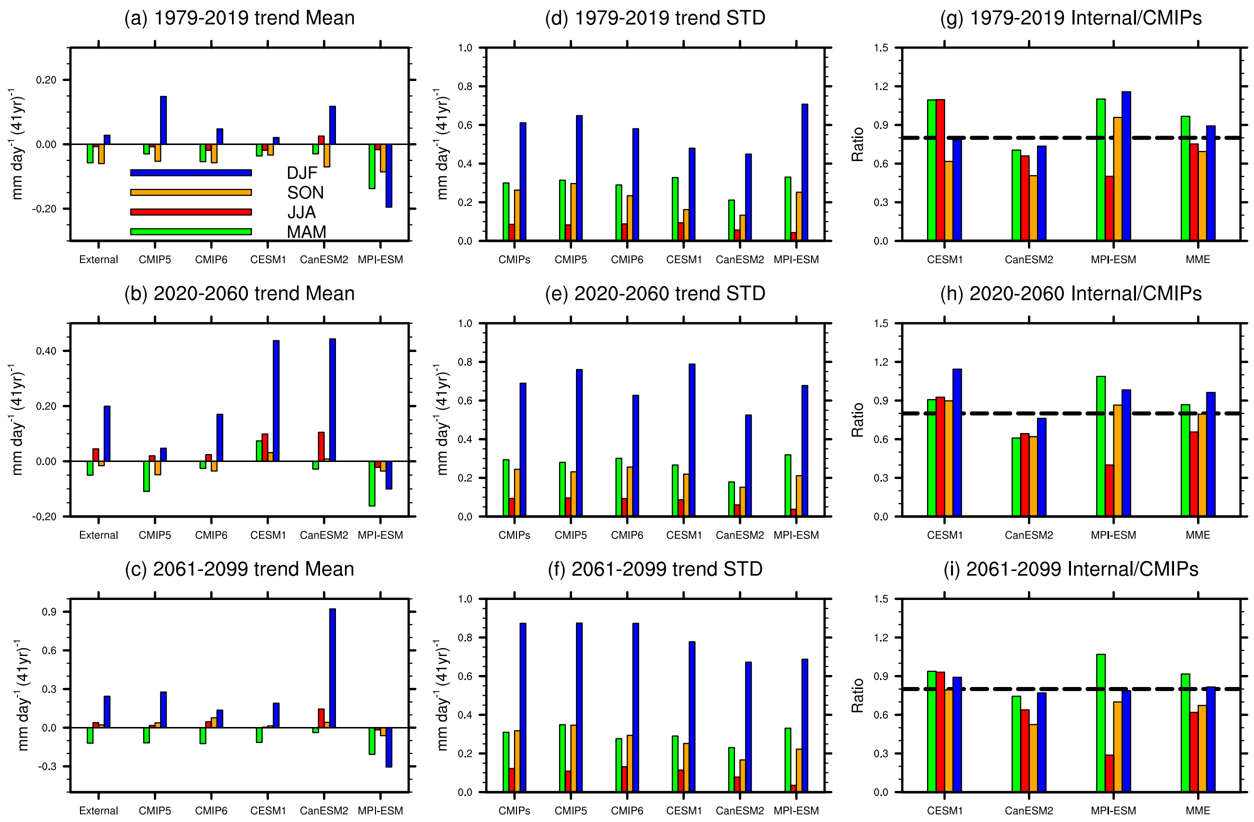

Supplementary Fig. 3 Precipitation trend averaged over California for the (a-c) mean signal, (d-f) standard deviation, (g-i) ratio of the uncertainty from internal variability relative to the uncertainty based on 57 CMIP5 and 71 CMIP6 members during 1979-2019 (first row), 2020-2060 (second row), 2061-2099 (third row). External denotes the effect of external forcing based on the average of the ensemble mean of CMIP5, CMIP6 and 3 large ensembles; CMIPs denotes the uncertainty based on a total of 128 CMIP5 and CMIP6 models (See Materials and Methods). Four seasons are calculated based on DJF (blue), SON (orange), JJA (red), and MAM (green). Units: mm day^{-1} 41year^{-1}

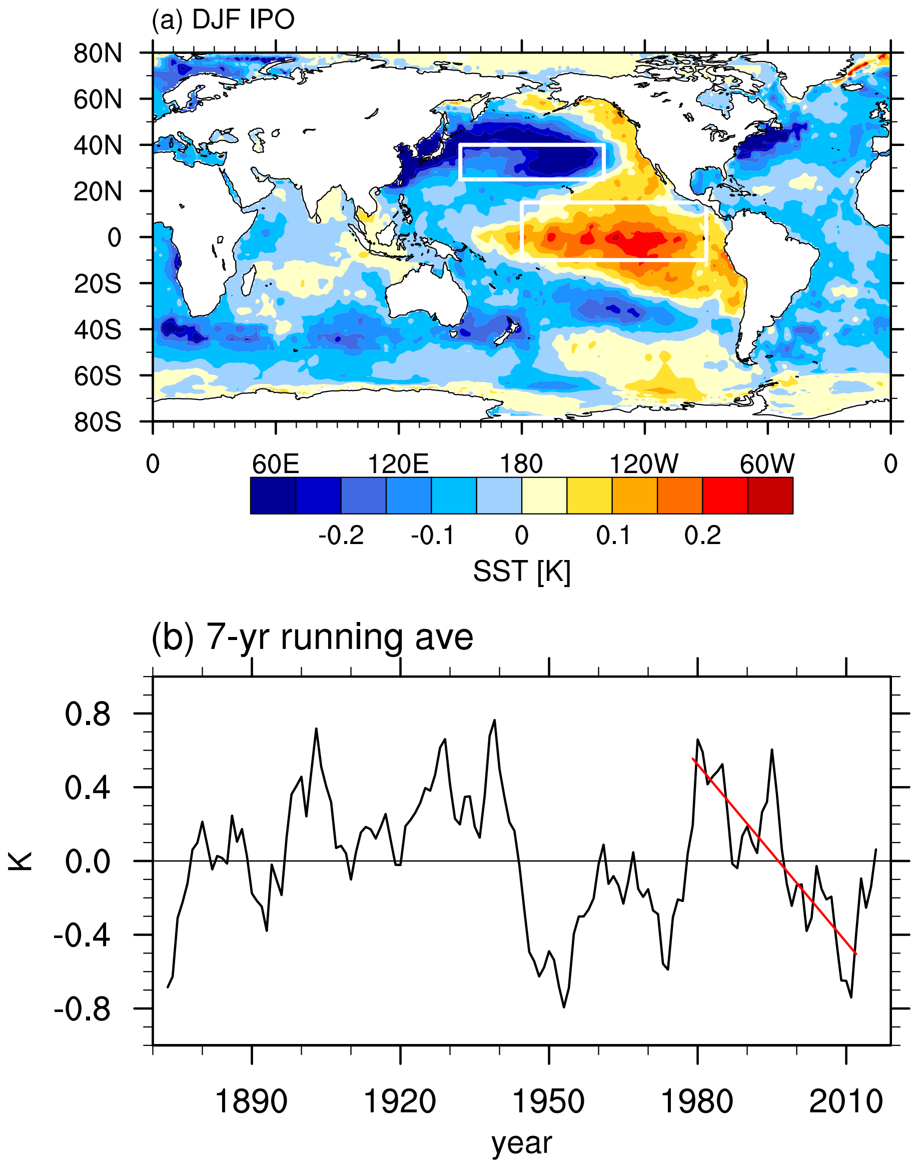

Supplementary Fig. 4 (a) Spatial pattern and (b) time series of the IPO in observation from HadISST. The IPO index is defined as the 7-year running average of the differences between SST anomalies averaged within the tropical central-eastern Pacific (180°E to 90°W, 10°S to 15°N) and the North Pacific (150°E to 140°W, 25°N to 40°N), respectively. The SST anomalies are obtained by deviations in each year from the long-term mean. The red line represents the linear trend during 1979-2019 of -1.0K $41year^{-1}$.

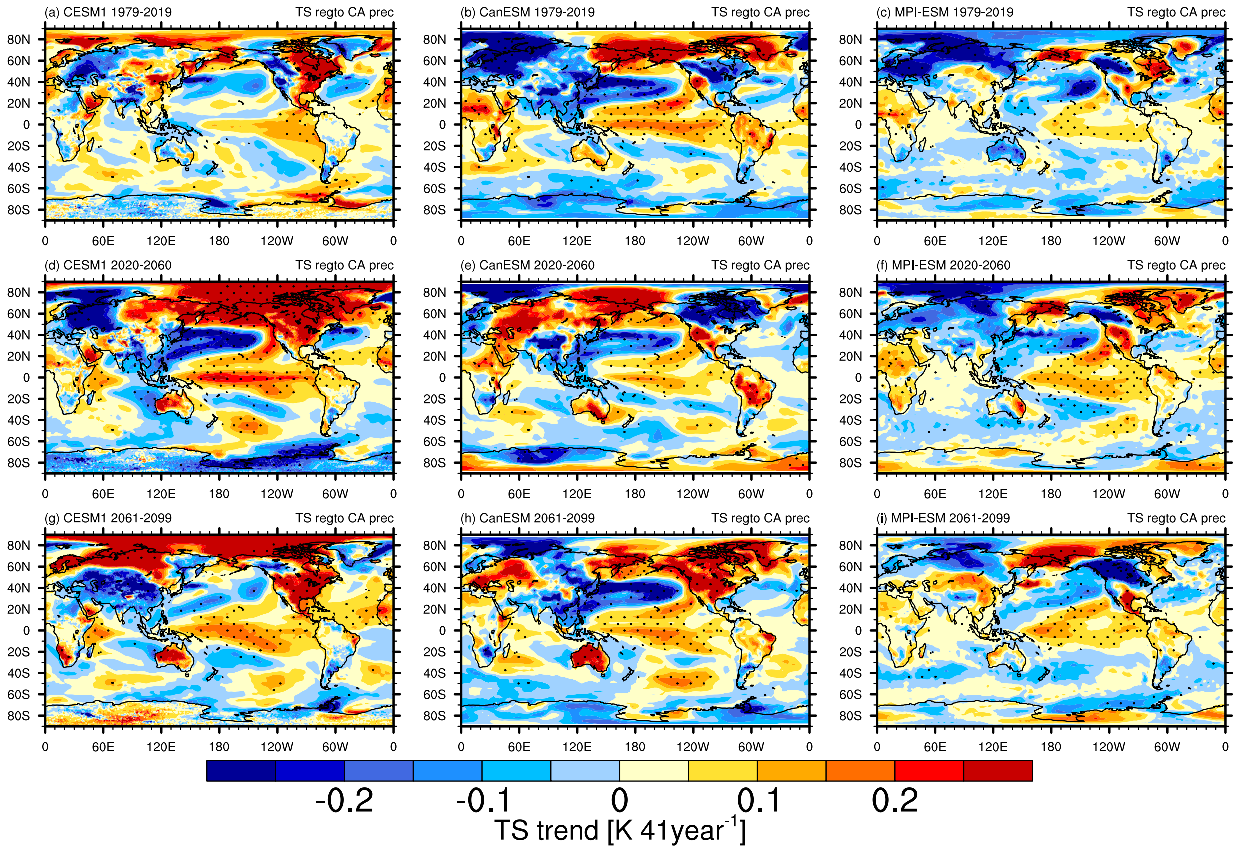

Supplementary Fig. 5 Inter-member regression of trends in surface temperature onto trends in California precipitation in winter based on 40 CESM1 members (first column), 50 CanESM2 members (second column), and 100 MPI-ESM members (third column) during (a-c) 1979-2019, (d-f) 2020-2060, and (g-i) MPI-ESM.

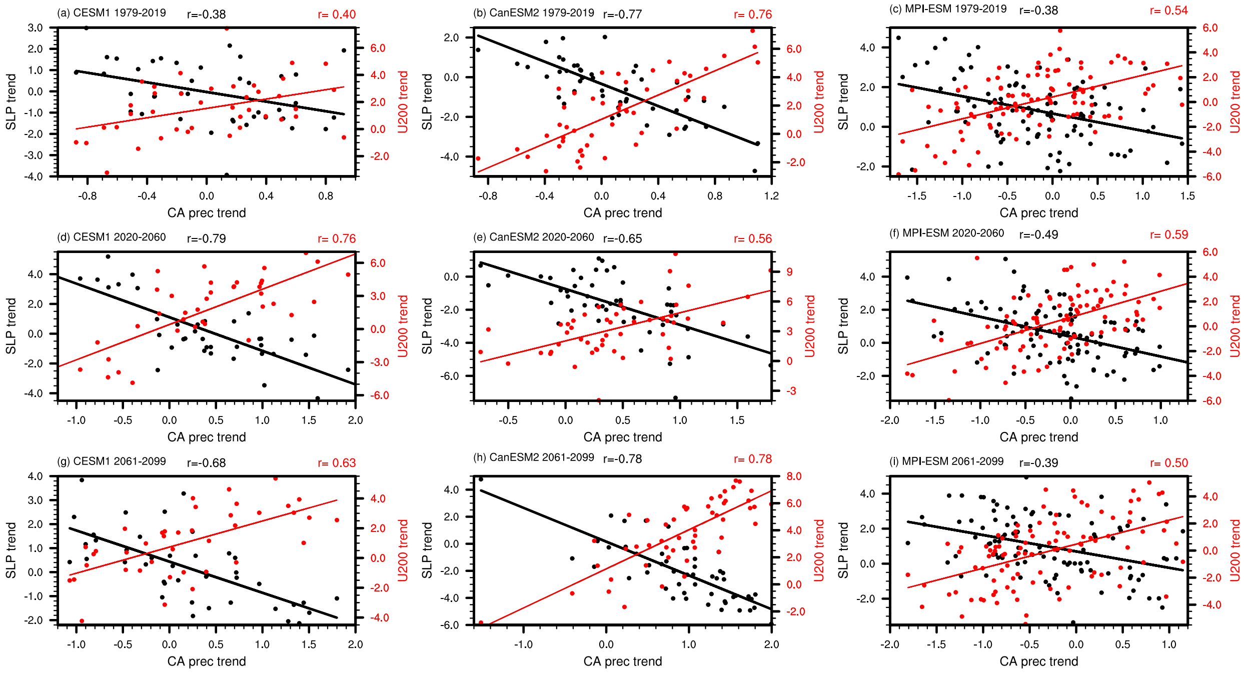

Supplementary Fig. 6 Scatterplots of the California winter precipitation trend (mm day^{-1} 41year^{-1}, x-axis), versus SLP trend over Aleutian low (hPa 41year^{-1}, black, left y-axis) and U200 trend over westerly jet extension (m s^{-1} 41year^{-1}, red, right y-axis) during (a-c) 1979-2019, (d-f) 2020-2060, (g-i) 2061-2099 based on 40 members of CESM1 (first column), 50 members of CanESM2 (second column), 100 members of MPI-ESM (third column). Regression lines and the inter-member correlations (r) are shown in corresponding color for each panel.

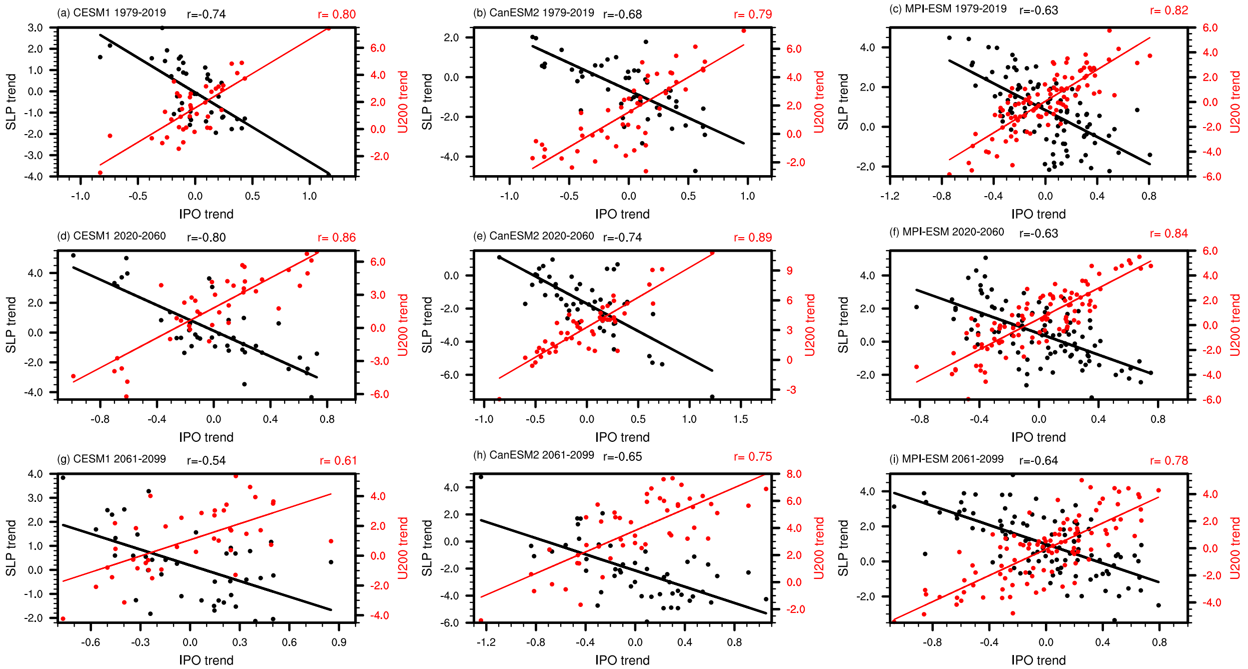

Supplementary Fig. 7 Same as Supplementary Fig. 6, but for the IPO trend (K 41year^{-1}) as x-axis.

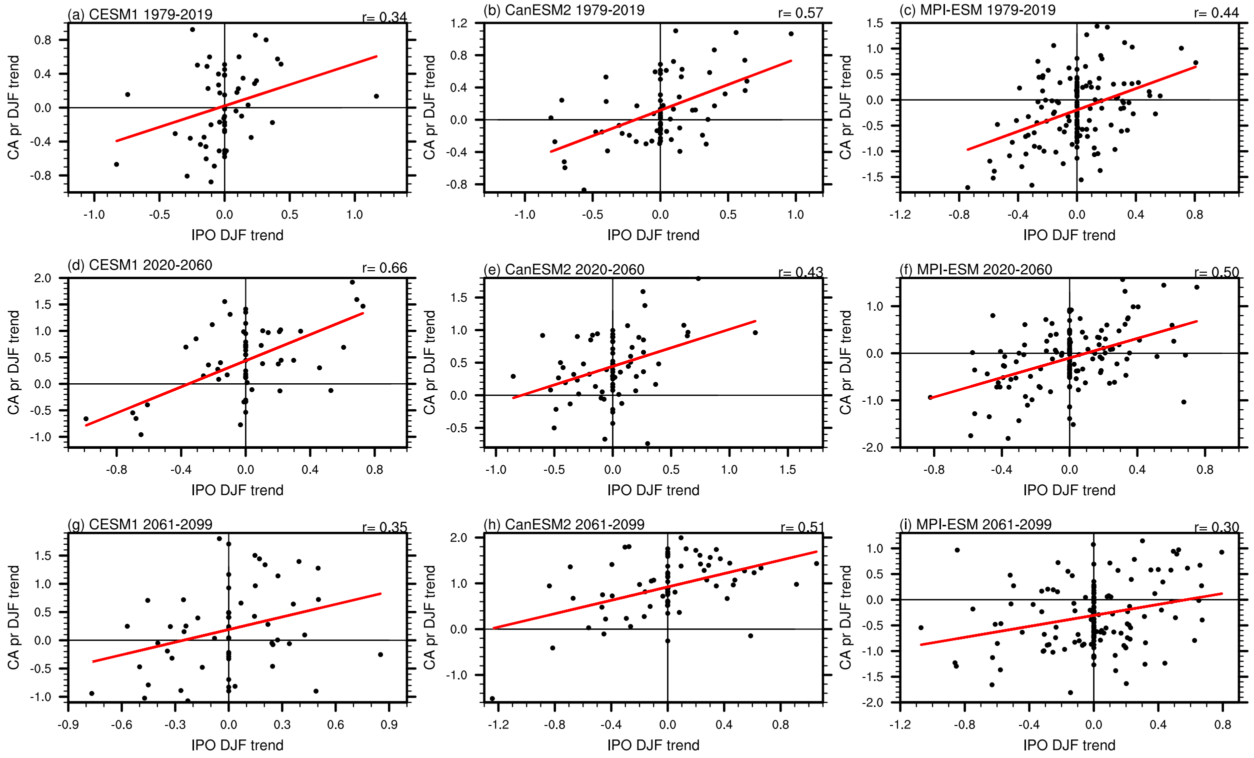

Supplementary Fig. 8 Scatterplots of the inter-member relationship between the IPO trends (K 41year^{-1}, x-axis) versus California precipitation trends (mm day^{-1} 41year^{-1}, y-axis) in winter during (a-c) 1979-2019, (d-f) 2020-2060, (g-i) 2061-2099 based on 40 members of CESM1 (first column), 50 members of CanESM2 (second column), 100 members of MPI-ESM (third column). Regression lines are shown as red line, and the inter-member correlations (r) are shown at the top-right of each panel.

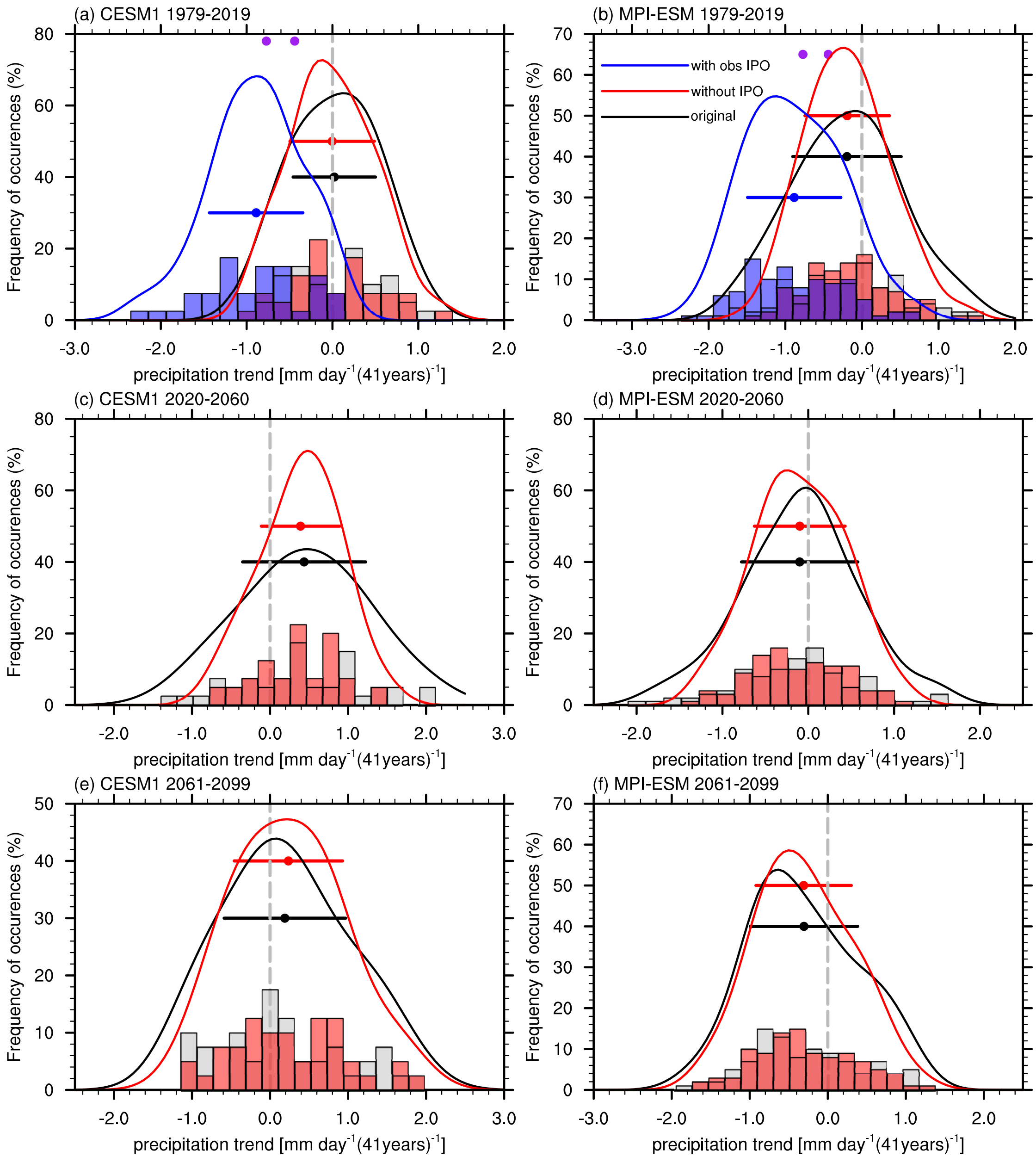

Supplementary Fig. 9 Histograms (bars) and 100-bins fitted distribution (lines) of the California winter precipitation trends during (a) 1979-2019, (b) 2020-2060 and (c) 2061-2099 based on 40 members of CESM1 and 100 members of MPI-ESM. The gray bars and the black fitted curves show the frequency of occurrence of the original trends; The red for those without the IPO's influence through linear regression against the IPO index in the individual runs; The blue for those including the observed IPO trend for 1979-2019. The dots and error bars denote the ensemble

mean and one STD of the distribution represented by the corresponding color. The purple dots denote observed precipitation trend based on GPCP (-0.44 mm day^{-1} 41year^{-1}) and CMAP (-0.77 mm day^{-1} 41year^{-1}).

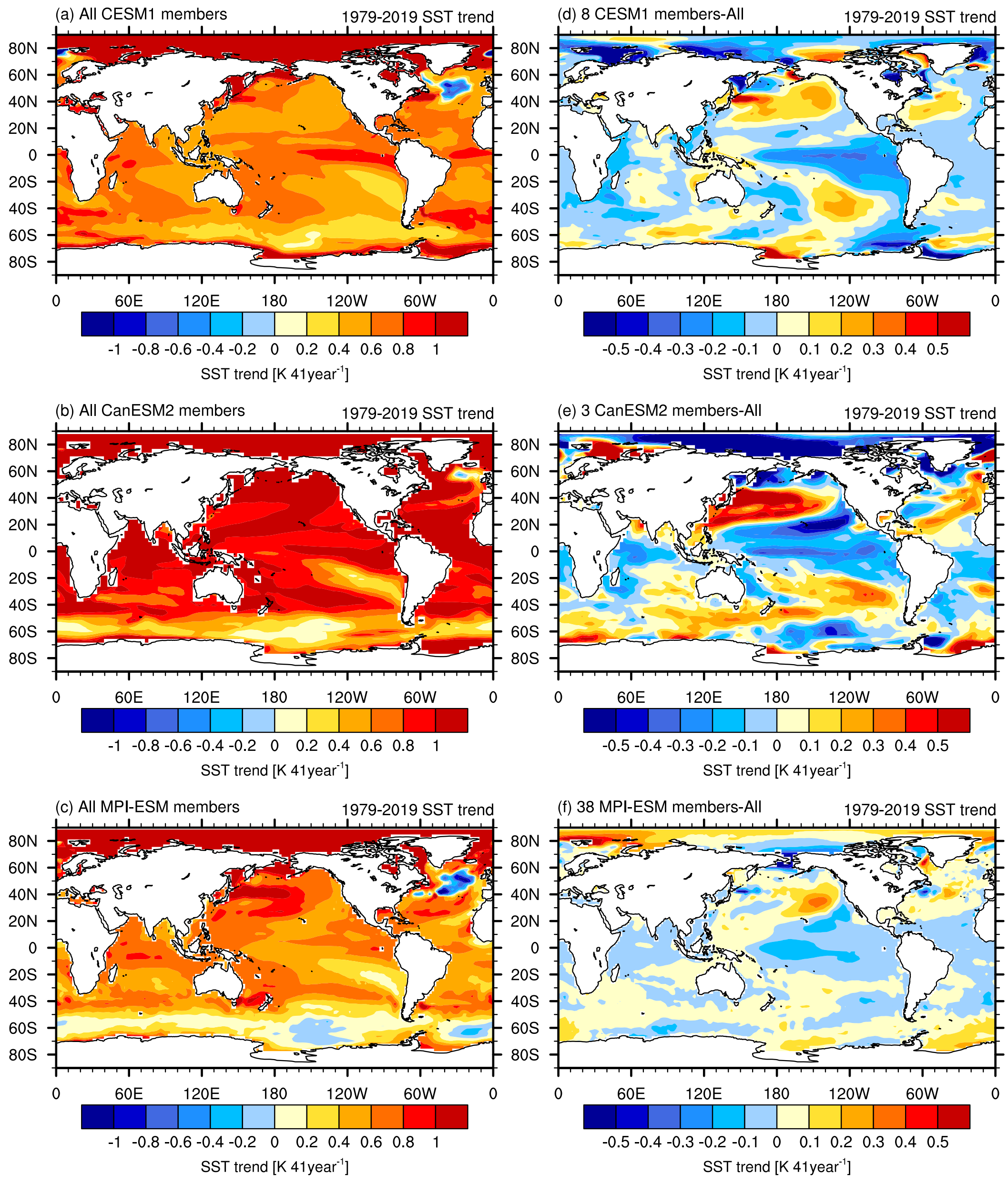

Supplementary Fig. 10 Composite of SST trends during 1979-2019 in winter based on (a) 40 CESM1 members, (b) 50 CanESM2 members, (c) 100 MPI-ESM members. Anomalies of composite SST trends for the members that can reproduce the observed drying CA stronger than the GPCP of -0.44 mm day^{-1} 41year^{-1} based on (d) 8 CESM1 members, (e) 3 CanESM2 members, (f) 38 MPI-ESM members, relative to all the members from the left column.

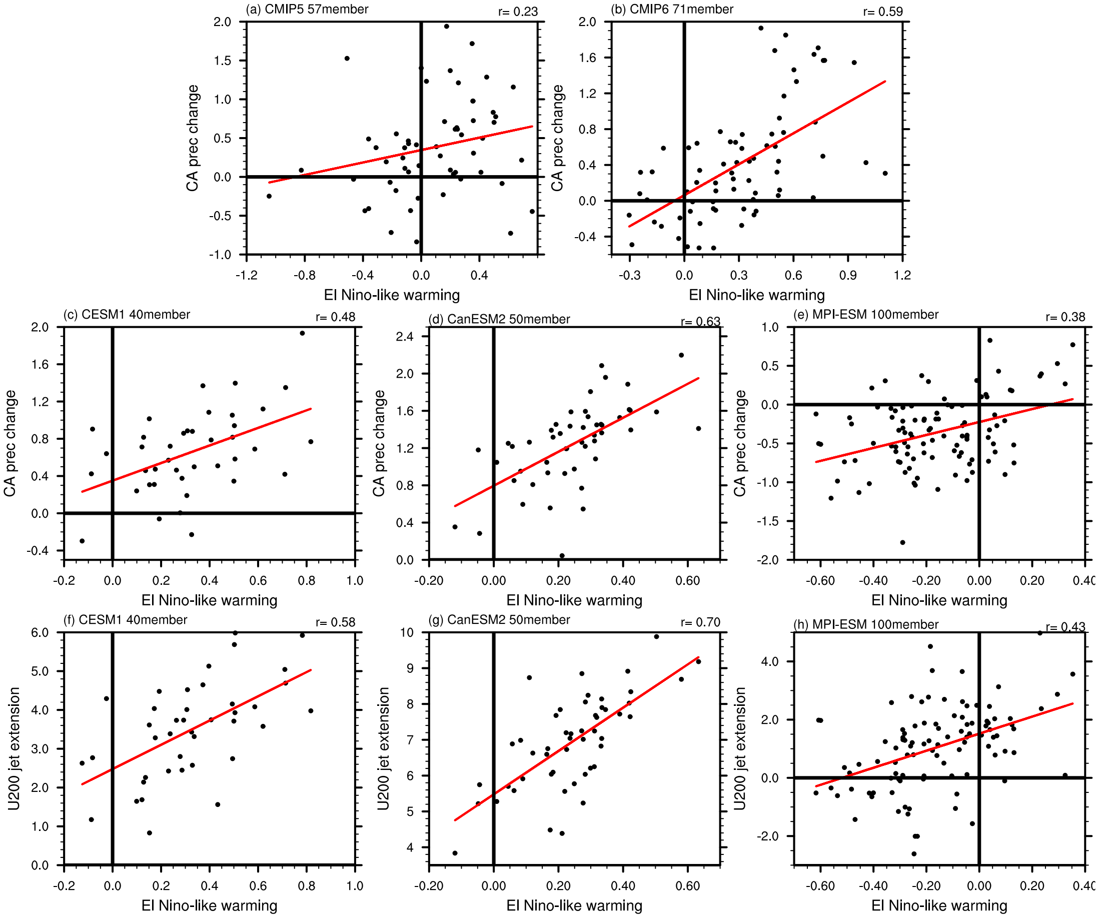

Supplementary Fig. 11 Scatterplots of the inter-member relationship between the El Niño-like pattern change (K, x-axis) versus California precipitation change (mm day^{-1}, y-axis) in winter based on (a) 57 members of 37 CMIP5 models, (b) 71 members of 37 CMIP6 models, (c) 40 members of CESM1, (d) 50 members of CanESM2, (e) 100 members of MPI-ESM. Scatterplots of the inter-member relationship between the El Niño-like pattern change (K, x-axis) versus 200hPa zonal wind change over westerly jet extension (m s^{-1}, y-axis) in winter based on (f) 40 members of CESM1, (g) 50 members of CanESM2, (h) 100 members of MPI-ESM. Regression lines are shown as red line, and the inter-member correlations (r) are shown at the top-right of each panel. All the changes are based on the difference between RCP8.5 (2085-2099) and Historical (1986-2000).

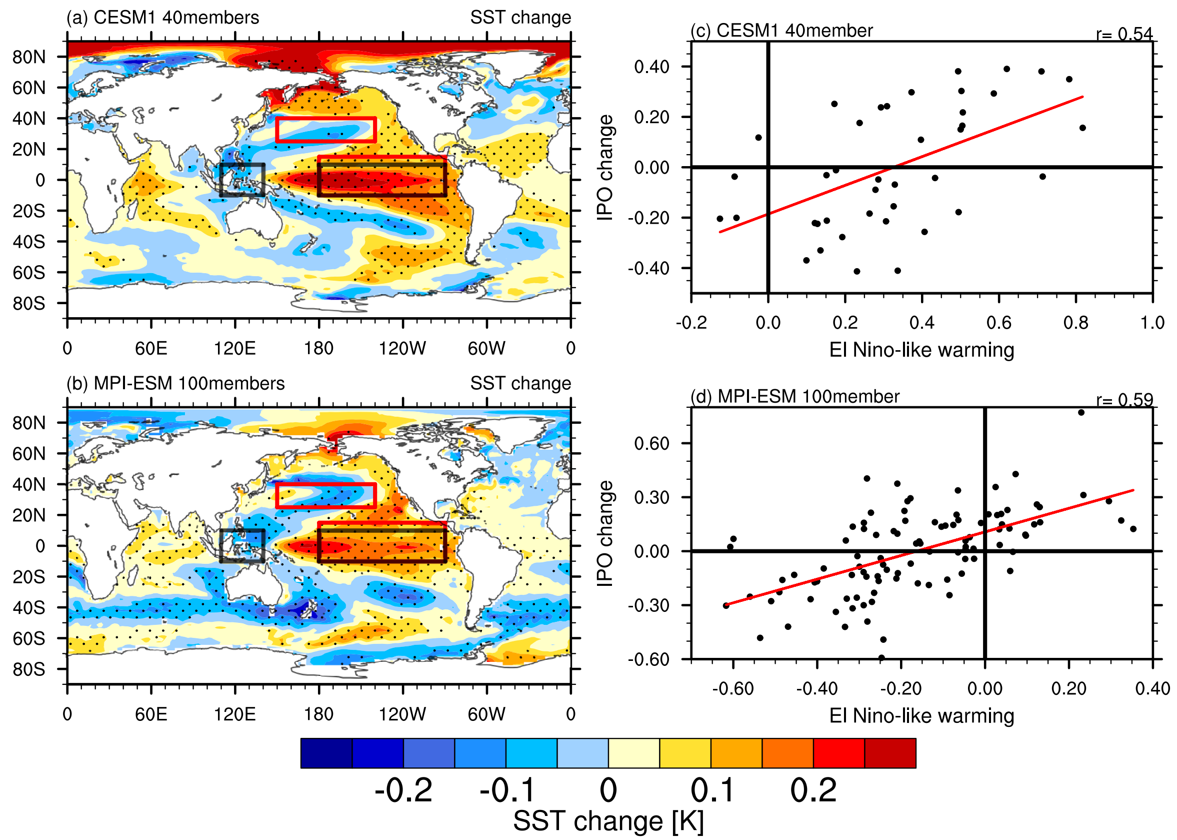

Supplementary Fig. 12 (a, b) Inter-member regressions of SST change (K) onto the El Niño-like pattern change, (c, d) Scatterplots of the El Niño-like pattern change (K, x-axis) versus the IPO change (K, y-axis) based on 40 members of CESM1 (upper column) and 100 members of MPI-ESM (lower column). Regression lines are shown as red lines and the inter-member correlations (r) are shown at the top-right of (c, d). El Niño-like pattern is based on the black rectangles, and the IPO is based on the red rectangles. All the changes are based on the difference between RCP8.5/SSP585 (2085-2099) and Historical (1986-2000).

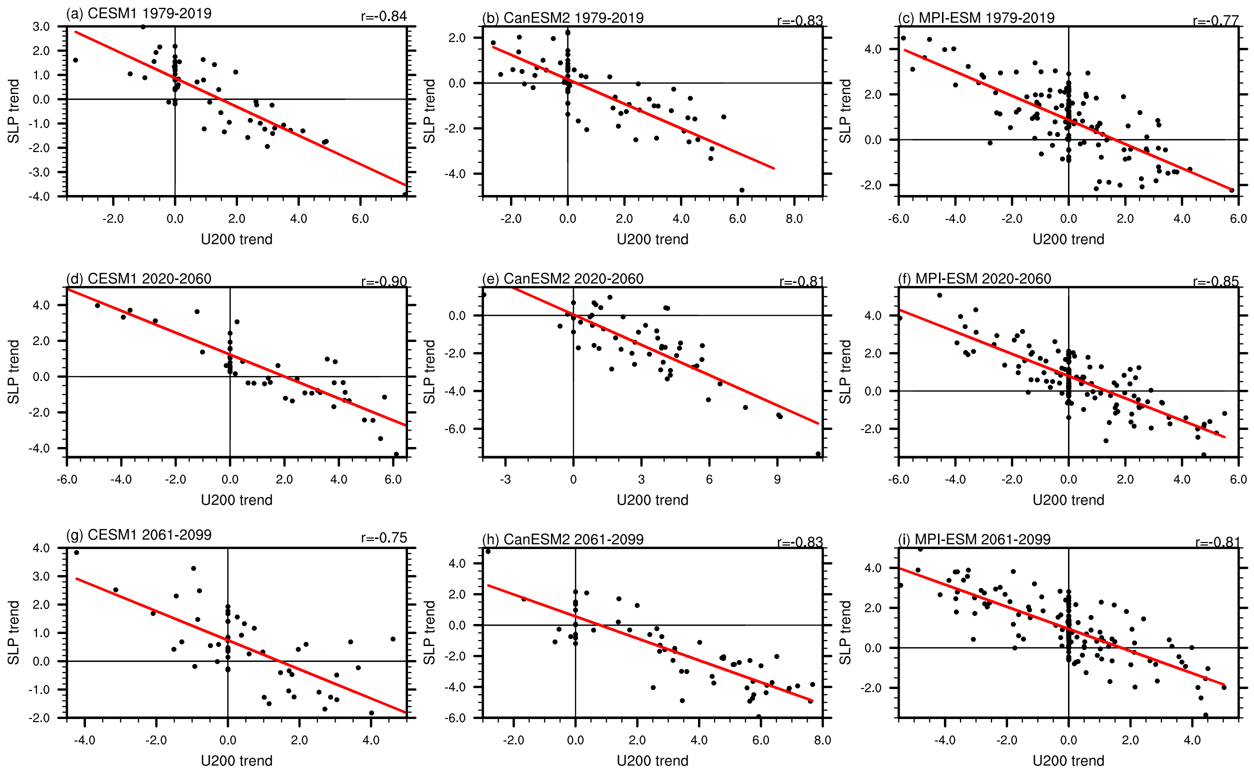

Supplementary Fig. 13 Scatterplots of the inter-member relationship between the U200 trend over westerly jet extension (m s^{-1} 41$year^{-1}$, x-axis) versus SLP trend over Aleutian low (hPa 41$year^{-1}$, y-axis) in winter during (a-c) 1979-2019, (d-f) 2020-2060, (g-i) 2061-2099 based on 40 members of CESM1 (first column), 50 members of CanESM2 (second column), 100 members of MPI-ESM (third column). Regression lines are shown as red line, and the inter-member correlations (r) are shown at the top-right of each panel.
